# The Gap between Estimated Incidence of End-Stage Renal Disease and Use of Therapy

**DOI:** 10.1371/journal.pone.0072860

**Published:** 2013-08-30

**Authors:** Shuchi Anand, Asaf Bitton, Thomas Gaziano

**Affiliations:** 1 Division of Nephrology, Department of Medicine, Stanford University School of Medicine, Stanford, California, United States of America; 2 Division of General Medicine, Brigham and Women’s Hospital, Harvard Medical School, Boston, Massachusetts, United States of America; 3 Cardiovascular Medicine, Brigham & Women’s Hospital, Harvard Medical School, Boston, Massachusetts, United States of America; Fundación para la Prevención y el Control de las Enfermedades Crónicas No Transmisibles en América Latina (FunPRECAL), Argentina

## Abstract

**Background:**

Relatively few data exist on the burden of end-stage renal disease (ESRD) and use of renal replacement therapy (RRT)–a life-saving therapy–in developing regions. No study has quantified the proportion of patients who develop ESRD but are unable to access RRT.

**Methods:**

We performed a comprehensive literature search to estimate use and annual initiation of RRT worldwide, and present these estimates according to World Bank regions. We also present estimates of survival and of etiology of diseases in patients undergoing RRT. Using data on prevalence of diabetes and hypertension, we modeled the incidence of ESRD related to these risk factors in order to quantify the gap between ESRD and use of RRT in developing regions.

**Results:**

We find that 1.9 million patients are undergoing RRT worldwide, with continued use and annual initiation at 316 and 73 per million population respectively. RRT use correlates directly (Pearson’s *r* = 0.94) with regional income. Hemodialysis remains the dominant form of RRT but there is wide regional variation in its use. With the exception of the Latin American and Caribbean region, it appears that initiation of RRT in developing regions is restricted to fewer than a quarter of patients projected to develop ESRD. This results in at least 1.2 million premature deaths each year due to lack of access to RRT as a result of diabetes and elevated blood pressure and as many as 3.2 million premature deaths due to all causes of ESRD.

**Conclusion:**

Thus, the majority of patients projected to reach ESRD due to diabetes or hypertension in developing regions are unable to access RRT; this gap will increase with rising prevalence of these risk factors worldwide.

## Introduction

The burden of end-stage renal disease (ESRD) is increasing across the world, propelled by the rising prevalence of two major risk factors: diabetes and hypertension [1]. Over the next two decades, the prevalence of hypertension and diabetes will rise disproportionately–by more than 80–100%–in economically developing countries, whereas the projected increase is between 20–50% for developed countries [2], [3]. A concomitant rise in ESRD incidence is anticipated [4], but data on the prevalence and incidence of ESRD as well as access to therapy in developing countries remain sparse.

Currently available ESRD registry data only capture information on patients who are able to access renal replacement therapy (RRT), not all those who develop ESRD. Furthermore, RRT is not a proxy for ESRD incidence and prevalence for two reasons: 1) a large proportion of patients who develop ESRD in low-income countries are never offered RRT and 2) a significant proportion of those receiving RRT are removed from therapy or die annually. Even counts of patients on RRT are not able to be rigorously collected due in part to poor documentation and lack of continuity within a fragmented network of both public and private providers [5]. Registries are rare. Current overviews of RRT in developing regions come mostly from industry distributed surveys, expert opinions, or extrapolations from decades-old data [6]–[8]. These reports rarely include data on the annual initiation rates of regional RRT, survival, or a breakdown of the underlying diseases among patients undergoing RRT.

Most importantly, current reports do not capture the large proportion of patients who die without ever accessing care. An assessment of RRT in developing regions would therefore underestimate the true burden of ESRD. Experts in India estimate that 10 percent of patients who reach ESRD are able to afford long-term RRT [5]. In a single-center report from South Africa, half of the patients with ESRD were unable to initiate government-sponsored treatment [9]. No study has attempted to systematically quantify this gap in care and its associated mortality.

In order to present a comprehensive picture of both treated ESRD (i.e. RRT) and to estimate the potential gap between actual incidence of ESRD and access to RRT, we performed a comprehensive literature search to present available data on the use and annual initiation of RRT, for all countries developed and developing, and grouped our estimates according to the seven World Bank regions. We also report available data on survival on each modality of RRT and the relative prevalence of three major diseases–diabetes, hypertension, and glomerulonephritis–among patients undergoing RRT. Finally, we estimate the gap between RRT and ESRD in developing countries by modeling the incidence of ESRD attributable to diabetes and hypertension.

## Methods

**Figure 1:**
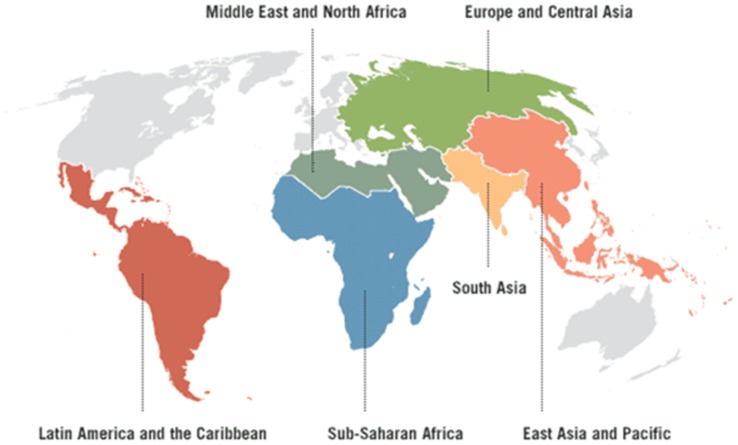
World Bank regions. Six of the seven World Bank regions are organized according to geography. The seventh region, the High Income region (shown here in white) includes North America, Japan, Taiwan, Australia, New Zealand, and European countries–based on a gross national income greater than $12 276 per capita in 2010. Figure adapted from World Bank Regions, Disease Control Priorities Project. www.dcp2.org/page/main/BrowseCountries.html.

Renal replacement therapy (RRT) is defined as any of hemodialysis, peritoneal dialysis, or kidney transplant. Our data are presented according to the following developing regions (as collated geographically by the World Bank): 1. Europe and Central Asia, 2. Latin America and Caribbean, 3. Middle East and North Africa, 4. East Asia and Pacific, 5. South Asia, and 6. Sub-Saharan Africa (Figure 1 **)**. Countries with Gross National Income greater than $12 276 per capita in 2010 were classified as “High Income” by the World Bank and are used as comparators in our analysis [10].

### Global use and Initiation of RRT

We used PUBMED and Google Scholar to search for regional registries and individual country data on RRT. To estimate incidence and prevalence of RRT, we employed key terms “renal registry (region, country)” and “transplant registry (region, country).” Whenever registries from an individual country or a regional organization were available, we used them as the predominant source (Appendix S1) [11]–[15]. For three of the seven World Bank regions–High Income, Europe and Central Asia, and Latin America and Caribbean–registries of RRT are available [16]–[20]. For regions or countries without registries, we used data cited in articles describing the state of RRT in a particular country. To collect these articles, we performed PUBMED and Google Scholar searches with the key terms “epidemiology of ESRD (region, country),” “renal replacement therapy (region, country),” “hemodialysis (region, country),” “peritoneal dialysis in (region, country),” and “renal transplants (region, country).” We included all articles starting from year 2000 up to year 2010. Some countries report data to the U.S. Renal Data System (USRDS) through an individual questionnaire [17]. If we found no recent data for a particular country through registries or our literature search, we used the data assembled by USRDS as the primary source. These countries were: Republic of Korea, Philippines, Taiwan, and Thailand. To estimate use and initiation rates at a regional level, we totaled mid-2005 population for each country and assigned a weight to each country’s population. For example, China’s population constituted 71% of the data within the East Asia and Pacific region; regional use of RRT was then a weighted average incorporating this proportion (see Appendix S2 for an example calculation). We examined the association between prevalent RRT and regional income, using data on gross national product (in purchasing power parity units) for each region.

### Estimates of ESRD in Patients with Diabetes and Hypertension

We estimated ESRD related to diabetes and hypertension in all individual developing countries. We first collected data on the prevalence of diabetes as well as the distribution of systolic blood pressure with mean and standard deviation, for adults over 20 years-old, using data collected on each of the 199 countries covered in the Global Burden of Metabolic Risk Factors of Chronic Diseases Collaborating Group [21], [22]. We then used the Multiple Risk Factor Intervention Trial (MRFIT), which is the only study that supplies prospective data on incidence of ESRD alone based on diabetes and blood pressure level–whereas the United Kingdom Prospective Diabetes Study (UKPDS) provides incidence of both elevated serum creatinine and ESRD combined [23]–[25]. The relative risk for ESRD among MRFIT participants with diabetes was 12.7 (95% confidence interval: 10.5 to 15.4) over a baseline risk of 13.7 per 100 000 patient-years among participants without diabetes (Appendix S3). The MRFIT reports that relative risk for ESRD increased by two-fold (95% confidence interval: 1.8 to 2.1) for each standard deviation increase in systolic blood pressure over a baseline risk of 5.3 per 100 000 patient-years among participants with optimal blood pressure. Using estimates of mean blood pressures and prevalence of diabetes from the global burden of disease (GBD) project, we estimated incidence of ESRD due to hypertension and diabetes. We then compared these rates to the annual rates of RRT initiated in each region to determine a *minimum* potential gap in ESRD treatment. We presumed that all patients comprising the gap between estimated incident ESRD and annual rates of RRT died prematurely.

For select countries in each of the regions, we were also able to assess the relative proportion of ESRD incidence attributed to blood pressure and diabetes in comparison to the other causes such as glomerulonephritis and interstitial nephritis. This allowed us to calculate a *maximum* potential gap in ESRD treatment.

### Survival by Modality and Etiology of Diseases in Patients Undergoing RRT

Sufficient data to create regional estimates for survival on RRT were not available through our literature search. We therefore present data for the most populous country in each region. Russia, Brazil, Egypt, China, India, and Nigeria represent the Europe and Central Asia, Latin America and Caribbean, Middle East and North Africa, East Asia and Pacific, South Asia and Sub-saharan Africa regions, respectively. For the relative proportion of patients on RRT with underlying diabetes, hypertension or glomerulonephritis as a cause of ESRD, we found regional estimates for Europe and Central Asia. In other developing regions, we present data from the most populous country within the region.

## Results

### Global use and Initiation of RRT

Our literature search yielded data from year 2000 or later for at least 70% of countries from each region (Figure 1), with the exception of Sub-Saharan Africa where incidence estimates are based on 30% of countries within the region. Roughly 1.9 million patients are undergoing RRT worldwide, yielding usage of 316 per million population (pmp) and annual initiation of 73 pmp (Table 1). About a third (648,000) resides in developing regions, which represent 85% of the world’s population.

**Table 1:** Use and annual initiation of renal replacement therapy.

World Bank Region	Use of RRT	Initiation of RRT
	(pmp)	(pmp)
Eastern Europe and Central Asia	332	79
Latin America and Caribbean	441	147
Middle East and North Africa	325	111
East Asia and Pacific	54	29
South Asia	33	10
Sub-Saharan Africa	20	10
High Income	1283	226
**All Regions Combined**	**316**	**73**

Data are presented according to World Bank Regions, with High Income countries as comparators.

**Abbreviations:** RRT–renal replacement therapy, pmp–per million population.

**Figure 2:**
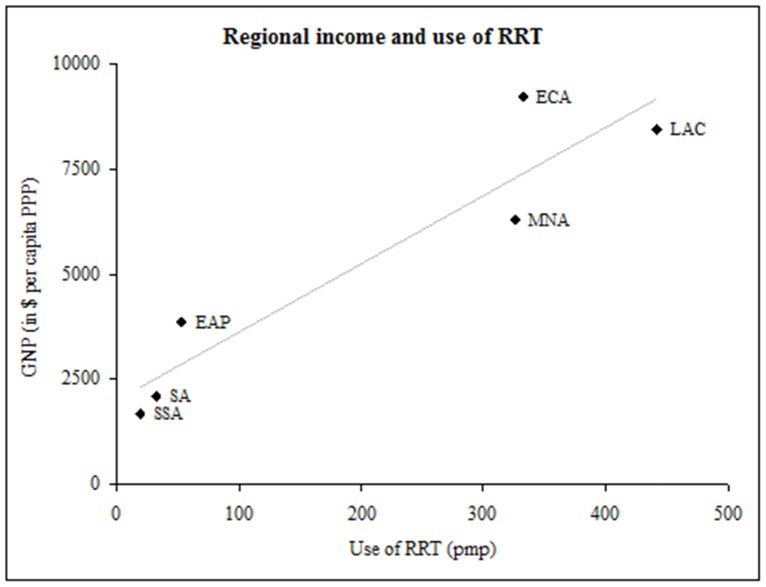
Regional income and use of RRT. Correlation between income and use of RRT in World Bank Regions (Pearson’s *r* = 0.94). Income is reported as GNP in U.S. dollars per capita PPP from 2005. Abbreviations: RRT-renal replacement therapy, GNP-gross national income, PPP-purchasing power parity, ECA-Eastern Europe and Central Asia, LAC-Latin America and Caribbean, MNA-Middle East and North Africa, EAP-East Asia and Pacific, SA-South Asia and SSA-Sub-Saharan Africa.

There is wide variation within and across regions in the fraction of the population starting and maintained on RRT. However, RRT use was directly correlated with *per capita* gross national product of each region (Figure 2, Pearson’s *r = *0.94). In Europe and Central Asia, Russia has the lowest numbers of patients getting RRT with use of 115 pmp and initiation rate of 20 pmp [18]. In Latin America and Caribbean, Puerto Rico has the highest RRT use at 1181 pmp; Nicaragua the lowest at 22 pmp [20]. In Middle East and North Africa, Lebanon has highest use of RRT at 818 pmp; Egypt the highest initiation rate at 200 pmp [6], [26]. In East Asia and Pacific, Malaysia has the highest use of RRT at 574 pmp, and Myanmar the lowest use at 3 pmp [11], [27]. In South Asia, Bangladesh reported the highest use of RRT at 83 pmp; Nepal the lowest at 22 pmp. In the Sub-Saharan region, the Democratic Republic of Congo and Uganda had the lowest use of RRT at less than one pmp, whereas South Africa had the highest at about 99 pmp [28].

**Figure 3:**
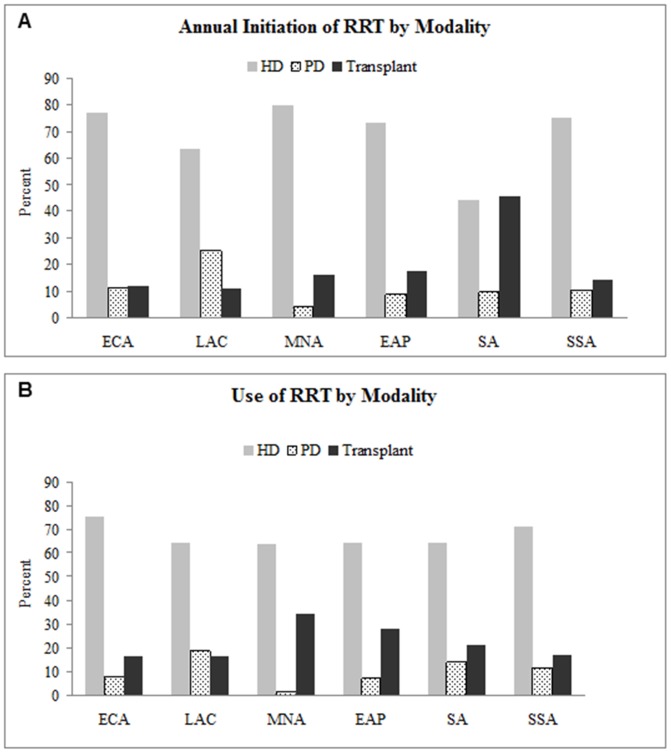
Annual initiation of RRT and use of RRT by modality. Hemodialysis is the most commonly used therapy within developing regions. Transplants are relatively more commonly used and initiated therapies in MNA and SA regions. Peritoneal dialysis is relatively more commonly used and initiated in LAC. Abbreviations: RRT-Renal replacement therapy, HD-Hemodialysis, PD-Peritoneal dialysis, ECA-Eastern Europe and Central Asia, LAC-Latin America and Caribbean, MNA-Middle East and North Africa, EAP-East Asia and Pacific, SA-South Asia and SSA-Sub-saharan Africa.

Although hemodialysis was the most commonly used modality of RRT, relative use of peritoneal dialysis and transplant also varied across regions (Figure 3). In Europe and Central Asia, 75% of prevalent and incident patients undergo hemodialysis. In Latin America and the Caribbean, hemodialysis is less frequently used compared to other regions (at 56% of all RRT). Peritoneal dialysis is the dominant modality in Mexico, El Salvador, Guatemala, Dominican Republic and Nicaragua. The Middle East and North Africa region uses hemodialysis primarily, but transplants compose nearly half of prevalent RRT in Saudi Arabia and Iran. In Sub-Saharan Africa, peritoneal dialysis is rarely used [28]. Kidney transplantation is only available in Cameroon, Mauritius, Kenya, Nigeria, South Africa and Sudan [29].

### Estimates of ESRD in Patients with Diabetes and Hypertension

**Figure 4:**
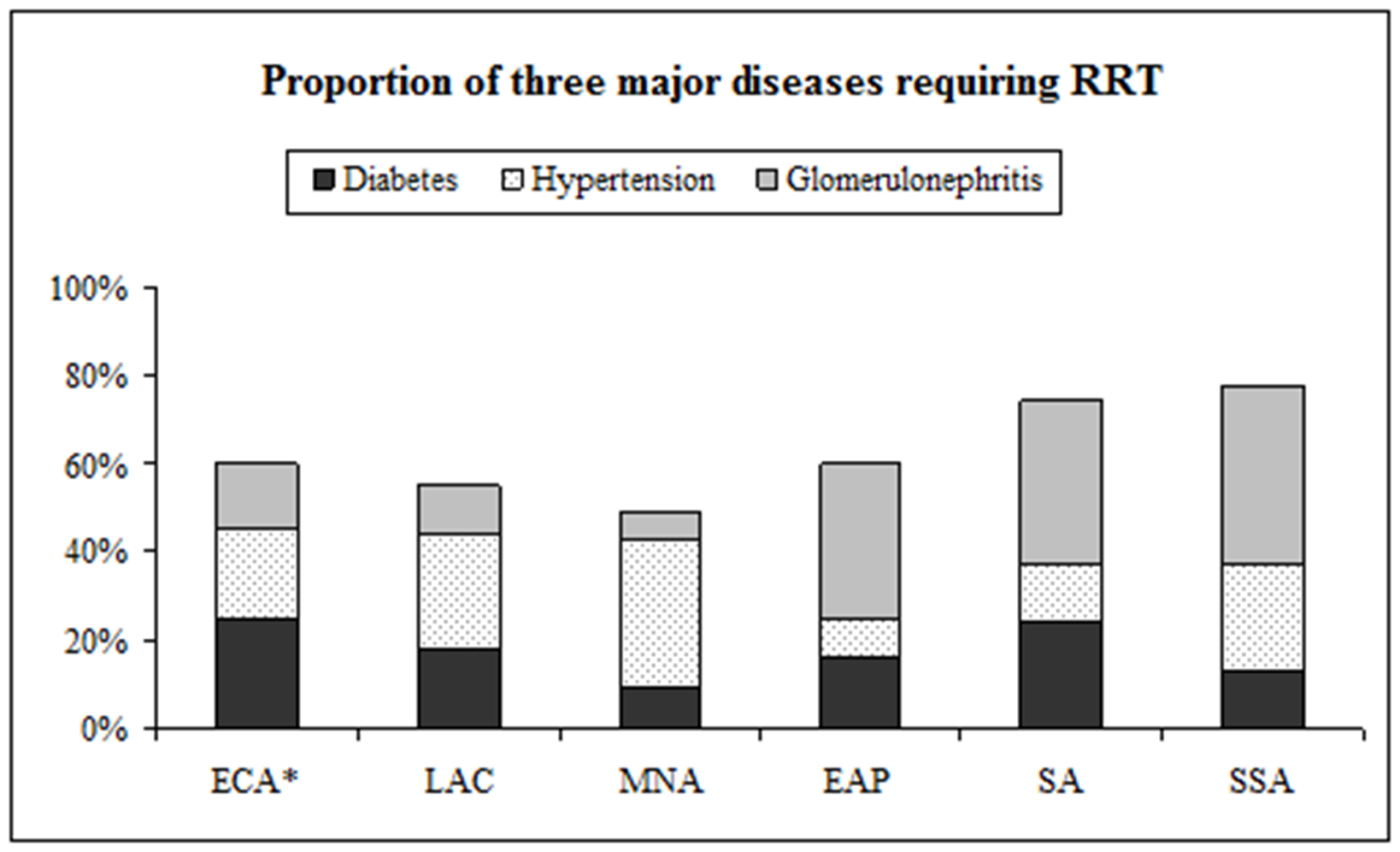
Proportion of diabetes, hypertension and glomerulonephritis among patients undergoing RRT. Data for ECA are from registry data representing 60% of its population; for all other developing regions, data are from the most populous country within the region. Abbreviations: HD-Hemodialysis, PD-Peritoneal dialysis, ECA-Eastern Europe and Central Asia, LAC-Latin America and Caribbean, MNA-Middle East and North Africa, EAP-East Asia and Pacific, SA-South Asia and SSA-Sub-Saharan Africa.

Tables 2 presents the projected ESRD incidence in patients with diabetes and hypertension, compared with the initiation of RRT for 147 countries aggregated by developing country region (individual country estimates are located in Appendix S3). Overall, we estimate that 1.2 million patients with diabetes or hypertension residing in developing countries are unable to access RRT annually. If we use current data on etiology of ESRD among patients undergoing RRT (i.e., that between 25–45% of patients on RRT have diabetes or hypertension in developing regions, Figure 4) and assume that this ratio reflects ESRD in the general population, we can estimate the number of patients attributable to all causes who are not able to access therapy to be closer to 3.2 million annually.

**Table 2:** Estimated incident ESRD and reported initiation of RRT in patients with diabetes and hypertension.

World Bank Region	Estimated incident ESRD in patients with diabetes	Estimated incident ESRD in patients with hypertension	Total estimated ESRD in patients with diabetes or hypertension	Estimated gap in access to therapy in patients with diabetes or hypertension
	(pmp)	(pmp)	(pmp)	
Eastern Europe and Central Asia	88	138	226	90,935
Latin America and Caribbean	91	118	209	77,794
Middle East and North Africa	82	114	196	49,729
East Asia and Pacific	146	165	311	450,091
South Asia	102	105	207	302,147
Sub-Saharan Africa	99	140	239	176,508

Incident ESRD is modeled using relative risk of ESRD in patients with diabetes and hypertension, as reported in the Multiple Risk Factor Intervention Trial [23], [24]. Sources for reported incident RRT in patients with diabetes and hypertension are noted in Appendix S3.

Abbreviations: ESRD–end-stage renal disease, RRT–renal replacement therapy.

The East Asia and Pacific region had the greatest expected incidence of ESRD related to diabetes and hypertension–at 311 pmp. The greatest gap between expected incidence of ESRD and actual initiation of RRT was also seen in this region, with 450,091 patients with diabetes or hypertension unable to access RRT annually, based on current data of RRT use. Fewer than five percent of patients projected to develop ESRD related to diabetes and hypertension access therapy in East Asia and Pacific (2.3%), South Asia (1.8%), and Sub-saharan Africa (1.5%). In Eastern Europe and Central Asia 15.7%, in Middle East and North Africa 23.8%, and in Latin America and Caribbean 30.9% of patients with diabetes and hypertension access therapy.

### Survival by Modality and Etiology of Diseases in Patients Undergoing RRT

Table 3 presents survival estimates for the most populous country in each region. With the exception of sub-Saharan Africa and South Asia, the most populous countries in each developing region reported first-year dialysis survival over 85% (compared with 79% in U.S.). Transplant survival was also reported as over 90% in developing regions (compared with 92% in the U.S.). Diabetes was the most common incident etiology requiring RRT in Europe and Central Asia at 29% of patients undergoing RRT (Figure 4). Hypertension was reported as the most common cause in Brazil (Latin America and Caribbean) and in Egypt (Middle East and North Africa), at 26% and 33% respectively. Glomerulonephritis was reported as the cause of ESRD in a third or more of patients undergoing RRT in East Asia and Pacific, Sub-Saharan Africa and South Asia.

**Table 3:** Estimates of first-year survival on renal replacement therapy.

World Bank Region	Representative country	One-year survival on dialysis (%)	One-year survival withtransplant (%)
		[Range of estimate*]	[Range of estimate*]
Eastern Europe and Central Asia	Russia	88 [80–94]	90 [90–98]
Latin America and Caribbean	Brazil	87 [75–87]	95 [91–98]
Middle East and North Africa	Egypt	97 [88–97]	95 [83–98]
East Asia and Pacific	China	89 [87–96]	98 [95–98]
South Asia	India	77 [65–90]	90 [85–94]
Sub-Saharan Africa	Nigeria	63 [63–90]	95 [78–95]

Survival is reported as survival on the therapy, for the most populous country in the World Bank Region.

* Ranges represent data from other countries within the region. Dialysis estimates are for hemodialysis and peritoneal dialysis.

## Discussion

Using a comprehensive literature search with emphasis on regional and individual country registries, we summarize the initiation and maintenance of RRT according to the seven World Bank regions, and estimate survival and the underlying etiology of ESRD among patients undergoing RRT. We also modeled the gap between anticipated ESRD incidence due to diabetes and hypertension, and the actual initiation of RRT in patients with diabetes and hypertension. The latter analysis demonstrates that in a majority of developing regions fewer than five percent of patients projected to develop ESRD related to diabetes and hypertension ever access RRT. Based on the population of adults living in developing regions, this translates into roughly 1.2 million patients with diabetes or hypertension dying prematurely due to lack of access to therapy and over 3 million premature deaths due to all causes of ESRD and lack of RRT.

We estimate a worldwide use of RRT of 316 pmp and annual initiation of 73 pmp. This translates into 1.9 million patients undergoing RRT in 2005, with 441 000 patients initiating RRT each year–on par with the expected growth in RRT from prior estimates. Using global data from 1990 and applying a growth rate of seven percent per year, Lysaught estimated that 1.1 million people were undergoing dialysis in 2001 [7]. More recently, through a questionnaire distributed to experts in 122 countries, Grassman et al. estimated that 1.8 million people were undergoing dialysis or living with kidney transplants at the end of 2004, with a growth rate of about six to seven percent per year [8].

We confirm also that a majority of countries rely primarily on hemodialysis, although there is some variability among and within regions. Our estimates for prevalent modality use–67% hemodialysis, 7% peritoneal dialysis and 25% transplant–are similar to those reported previously [8]. We extend current literature by tabulating these measures for initiation of RRT: 76% for hemodialysis and 12% each for peritoneal dialysis or transplant. As expected relative use of both hemodialysis and peritoneal dialysis drops after the first year as some patients transfer to the transplant population. However this drop is greater among patients on peritoneal dialysis, suggesting that a sizable proportion on peritoneal dialysis either receive kidney transplants or switch to hemodialysis after one year.

Unlike earlier reports, we also quantify the gap between incidence of *treated* ESRD and the anticipated incidence of all (treated and untreated) ESRD related to diabetes and hypertension in developing regions. Our model accurately projected ESRD incidence in patients with hypertension, and underestimated ESRD incidence for patients with diabetes, relative to actual counts available from USRDS. Using this model, we find that in a majority of developing regions, initiation rates of RRT fall below twenty-five percent of the projected incident ESRD related to diabetes or hypertension. Initiation rates of RRT track more closely with the income rather than with the prevalence of risk factors for ESRD. For example, the prevalence of diabetes among adults in India and Egypt is close to that of the U.S. but initiation of RRT among these patients falls well below the U.S. Similarly, the prevalence of hypertension in Nigeria (Sub-Saharan Africa) is higher than reported in India and China, but RRT initiation is much lower.

One exception to the association between gross national product and use of RRT was Eastern Europe and Central Asia. Despite similar per capita income, and projections of hypertension and diabetes-related ESRD, the Eastern European and Central Asian countries had lower initiation rates of RRT compared with Latin America and Caribbean countries. We speculate that differences in RRT rates may reflect the Brazilian government’s policy of fully reimbursing RRT even for patients using privatized facilities as well as the greater use of peritoneal dialysis in many Latin American countries.

In many regions, RRT-associated survival was actually higher than among countries classified as “High Income.” For transplants, one explanation lies in the expected graft survival: in the High Income region deceased donor transplants predominate, whereas in the developing regions living donor transplants predominate [30]. Patients with significant comorbidities are less likely to be accepted into RRT programs in developing regions. In South Africa where government-sponsored RRT is offered to patients who fulfill criteria for eventual transplantation, patients over 60 years of age and patients with diabetes were significantly less likely to be on RRT [9]. Thus, higher survival rates may reflect the better underlying health status of patients selected for RRT in developing regions. Glomerulonephritis was the most common etiology of ESRD among patients undergoing RRT in East Asia, South Asia and Sub-Saharan Africa. Again, whether this reflects a true difference in underlying etiology of ESRD in these regions, or the disproportionate selection of patients with glomerulonephritis (who tend to be considerably younger in age and have fewer comorbidities than patients with diabetes or hypertension/vascular disease) is unclear.

A higher proportion of patients on RRT were using peritoneal dialysis in the Latin America and Caribbean region than in other regions (23% compared with 6% in High Income region). Preference for peritoneal dialysis in Latin America–Mexico in particular–may reflect a purposeful strategy to deal with the shortage of nephrologists and dialysis centers. Historically, leading Mexican clinicians trained in peritoneal dialysis and disseminated the technique early [31].Rates of peritonitis and survival observed in Mexico are similar to that of the U.S. and other High Income countries [32]. A similar approach could be adopted in other developing countries to help increase access to RRT [33].

Two developing regions–Latin America and Caribbean, and Middle East and North Africa–provide RRT for comparatively greater proportion of patients. However, resources allocated to RRT in these regions are not likely to be sustainable, and may be unrealistic targets for other regions. The Brazilian Ministry of Health spent U.S. $500 million on RRT in 2004 [34]; in Egypt fully 28% ($100 million) of the health care budget was spent on government-sponsored RRT in 2008 [35]. The cost of peritoneal dialysis in developing regions is not necessarily lower than that of hemodialysis, largely due to the expense of importing solutions and lack of governmental policies that support high-volume peritoneal dialysis centers (with the exception of Mexico) [33]. Transplantation activity remains predominantly reliant on live organ donation. Thus, with the projected growth in ESRD, these regions are also likely to face additional challenges in financing care for larger groups of prospective patients.

Our study has several strengths. We provide updated estimates of RRT use for new and existing patients. In addition we provide estimates for survival and relative prevalence of three major etiologies for ESRD among patients undergoing RRT. Organization of our data according to World Bank regions allows a comparison between the prevalence of risk factors, regional income and use of RRT. Most importantly, we used a novel approach–modeling projected rates of incident ESRD based on available estimates of diabetes and hypertension prevalence–to provide a better approximation of the underlying rates of ESRD in each region. This allows us to explore the gap between probable “true” ESRD incidence and treated ESRD incidence recorded in registries and other sources.

An important limitation of this approach is that it assumes the progression to ESRD among patients with diabetes and hypertension will be similar to that seen in the U.S. Progression rates vary among different populations, with African Americans at higher risk for ESRD due to hypertension, for example [36]. Similarly Hallan et al. have demonstrated that although prevalence of CKD in Norway approaches that of the U.S., Norwegians are less likely to progress to ESRD, arguably due to a lower prevalence of obesity and earlier referral to nephrologists [37]. Thus, more population-specific models regarding rates of progression and competing risks for mortality are required to refine future projections. In addition our estimates are limited to patients with diabetes or hypertension. Our model *does* capture the two risk factors most likely to drive global ESRD incidence over the next two decades. The rapidly rising prevalence of diabetes and hypertension has been documented worldwide, and with improving survival from communicable diseases, many more patients will survive to develop ESRD. The gaps in care we have identified between actual and treated ESRD in most developing regions will only grow larger unless comprehensive approaches to CKD surveillance and management are adopted. Such policies will require careful deliberation and prioritization among politicians, health policy-makers, and individuals within each country or region.

## Supporting Information

Appendix S1 **Dialysis and transplant registries available and used predominantly.**
(DOCX)

Appendix S2 **All countries searched according to World Bank region and the proportion of available data within each region.**
(DOCX)

Appendix S3 **Individual country data for incident ESRD in patients with diabetes and hypertension.**
(DOCX)
