# Supplementary material for: The Gap between Estimated Incidence of End-Stage Renal Disease and Use of Therapy
Source: PLoS One. 2013 Aug 30;8(8):e72860. doi: 10.1371/journal.pone.0072860 (PMC3758352; doi:10.1371/journal.pone.0072860)
Supplement: Appendix S1 — Dialysis and transplant registries available and used predominantly. (DOCX) [file pone.0072860.s001.docx]

**Appendix S1: Dialysis and Transplant Registries available and used predominantly**

- **ANZ:** The Australian New Zealand Dialysis and Transplant Registry captures data reported from all renal dialysis and transplant units in the two countries. [www.**anz**data.org.au](http://www.anzdata.org.au)
- **CORR:** The Canadian Organ Replacement Registry publishes its annual report collected from hospital and independent dialysis programs and organ procurement organizations at <http://www.cihi.ca/cihiweb/dispPage.jsp?cw_page=services_corr_e>

The 2007 report presents data from 1996-2005.

- **Egyptian Society of Nephrology and Transplantation**. Its annual report for 2004 is available from: [www.esnonline.net/registry.php](http://www.esnonline.net/registry.php).
- **ERA-EDTA:** The European Renal Association and European Dialysis Transplant Association has established links with national and large regional registries in Europe. [www.era-edta-reg.org](http://www.era-edta-reg.org).
- **Malaysian NRR:**  The Malaysian National Renal Registry compiles an annual report based on voluntary submissions from participating hemodialysis, peritoneal dialysis and transplant centers. [www.msn.org.my/nrr](http://www.msn.org.my/nrr).
- **The Kidney Foundation of Pakistan**. Its 2005-2006 dialysis registry report gathers data from 195 centers and is available from [www.thekidneyfoundation.net.pk](http://www.thekidneyfoundation.net.pk).
- **Saudi Arabia Center for Organ Transplantation**. Available from: [www.scot.org.sa/annual-report](http://www.scot.org.sa/annual-report).
- **SLANH**: The Latin American Society of Nephrology (SLANH) has published annual reports detailing the state of renal replacement therapy (RRT) in the region. All twenty countries in the region are members of the society but submissions are voluntary.
- **Singapore Renal Registry:** 1999-2000 report available at [www.hpb.gov.sg/data/hpb.home/files/edu/srr_**report**_1999_2000_v4.pdf](http://www.hpb.gov.sg/data/hpb.home/files/edu/srr_report_1999_2000_v4.pdf) represents data on 95% of all dialysis and transplant patients in Singapore. This report published in 2007.
- **USRDS:** The US renal data system collects data from Medicare claims and a medical evidence report filed by renal practitioners. Annual reports are available at [www.usrds.org/](http://www.usrds.org/). We used 2005 data.
  - International data available through the USRDS

| Australia |
| --- |
| Austria |
| Bangladesh |
| Belgium, Dutch speaking |
| Belgium, French speaking |
| Bosnia & Herzegovina |
| Canada |
| Chile |
| Croatia |
| Czech Republic |
| Denmark |
| Finland |
| France |
| Germany |
| Greece |
| **Hong Kong** |
| Hungary |
| Iceland |
| Israel |
| Italy |
| Jalisco (Mexico) |
| Japan |
| **Rep. of Korea** |
| Luxembourg |
| Malaysia |
| Netherlands |
| New Zealand |
| Norway |
| Pakistan |
| **Philippines** |
| Poland |
| Russia |
| Scotland |
| Shanghai |
| Spain |
| Sweden |
| **Taiwan** |
| **Thailand** |
| Turkey |
| U.K., England, Wales & N Ireland |
| United States |
| Uruguay |

***Bolded countries represent countries whose USRDs submitted data was used in the current analysis**
