# Supplementary material for: The Gap between Estimated Incidence of End-Stage Renal Disease and Use of Therapy
Source: PLoS One. 2013 Aug 30;8(8):e72860. doi: 10.1371/journal.pone.0072860 (PMC3758352; doi:10.1371/journal.pone.0072860)
Supplement: Appendix S2 — All countries searched according to World Bank region and the proportion of available data within each region. (DOCX) [file pone.0072860.s002.docx]

**Appendix S2. All countries searched according to World Bank Region, and the proportion of available data within each region**

| **World Bank Region** | **Countries in Region** | **Countries with Available Data on RRT** | **Percent of Region Represented** |
| --- | --- | --- | --- |
| East Asia and Pacific | \| American Samoa \| \| --- \| \| Cambodia \| \| China \| \| Fiji \| \| Indonesia \| \| Kiribati \| \| Korea, Dem. Rep. \| \| Lao PDR \| \| Malaysia \| \| Marshall Islands \| \| Micronesia, Fed. Sts. \| \| Mongolia \| \| Myanmar \| \| Palau \| \| Papua New Guinea \| \| Philippines \| \| Samoa \| \| Solomon Islands \| \| Thailand \| \| Timor-Leste \| \| Tonga \| \| Vanuatu \| \| Vietnam \| | \| China \| \| --- \| \| Malaysia \| \| Thailand \| \| Philippines \| \| Vietnam \| | 78% |
| Europe and Central Asia | \| Albania \| \| --- \| \| Armenia \| \| Azerbaijan \| \| Belarus \| \| Bosnia and Herzegovina \| \| Bulgaria \| \| Croatia \| \| Czech Republic \| \| Estonia \| \| Georgia \| \| Hungary \| \| Isle of Man \| \| Kazakhstan \| \| Kyrgyz Republic \| \| Latvia \| \| Lithuania \| \| Macedonia, FYR \| \| Moldova \| \| Poland \| \| Romania \| \| Russian Federation \| \| Slovak Republic \| \| Tajikistan \| \| Turkey \| \| Turkmenistan \| \| Ukraine \| \| Uzbekistan \| \| Yugoslavia, Fed. Rep. \| | \| Bosnia-Herzegovina \| \| --- \| \| Bulgaria \| \| Croatia \| \| Czech Republic \| \| Estonia \| \| FYR of Macedonia \| \| Hungary \| \| Latvia \| \| Poland \| \| Romania \| \| Russia \| \| Slovakia \| \| Turkey \| \| Yugoslavia \| | 70% |
| Latin America and Caribbean  Regional | \| Antigua and Barbuda \| \| --- \| \| Argentina \| \| Barbados \| \| Belize \| \| Bolivia \| \| Brazil \| \| Chile \| \| Colombia \| \| Costa Rica \| \| Cuba \| \| Dominica \| \| Dominican Republic \| \| Ecuador \| \| El Salvador \| \| Grenada \| \| Guatemala \| \| Guyana \| \| Haiti \| \| Honduras \| \| Jamaica \| \| Mexico \| \| Nicaragua \| \| Panama \| \| Paraguay \| \| Peru \| \| Puerto Rico \| \| St. Kitts and Nevis \| \| St. Lucia \| \| St. Vincent and the Grenadines \| \| Suriname \| \| Trinidad and Tobago \| \| Uruguay \| \| Venezuela, RB \| | \| Argentina \| \| --- \| \| Bolivia \| \| Brazil \| \| Chile \| \| Colombia \| \| Costa Rica \| \| Cuba \| \| Dominican Republic \| \| Ecuador \| \| El Salvador \| \| Guatemala \| \| Honduras \| \| Mexico \| \| Nicaragua \| \| Panama \| \| Paraguay \| \| Peru \| \| Puerto Rico \| \| Uruguay \| \| Venezuela, RB \| | 97% |
| Middle East and North Africa | \| Algeria \| \| --- \| \| Djibouti \| \| Egypt, Arab Rep. \| \| Iran, Islamic Rep. \| \| Iraq \| \| Jordan \| \| Lebanon \| \| Libya \| \| Malta \| \| Morocco \| \| Oman \| \| Saudi Arabia \| \| Syrian Arab Republic \| \| Tunisia \| \| West Bank and Gaza \| \| Yemen, Rep. \| |  |  |
| South Asia | \| \| Afghanistan \| \| --- \| \| Bangladesh \| \| Bhutan \| \| India \| \| Maldives \| \| Nepal \| \| Pakistan \| \| Sri Lanka \| \| \| --- \| --- \| --- \| --- \| --- \| --- \| --- \| --- \| --- \| \|  \| |  |  |
| Sub-Saharan Africa | \| Angola \| \| --- \| \| Benin \| \| Botswana \| \| Burkina Faso \| \| Burundi \| \| Cameroon \| \| Cape Verde \| \| Central African Republic \| \| Chad \| \| Comoros \| \| Congo, Dem. Rep. \| \| Congo, Rep. \| \| Côte d'Ivoire \| \| Equatorial Guinea \| \| Eritrea \| \| Ethiopia \| \| Gabon \| \| Gambia, The \| \| Ghana \| \| Guinea \| \| Guinea-Bissau \| \| Kenya \| \| Lesotho \| \| Liberia \| \| Madagascar \| \| Malawi \| \| Mali \| \| Mauritania \| \| Mauritius \| \| Mayotte \| \| Mozambique \| \| Namibia \| \| Niger \| \| Nigeria \| \| Rwanda \| \| São Tomé and Principe \| \| Senegal \| \| Seychelles \| \| Sierra Leone \| \| Somalia \| \| South Africa \| \| Sudan \| \| Swaziland \| \| Tanzania \| \| Togo \| \| Uganda \| \| Zambia \| \| Zimbabwe \| |  |  |
| High Income | \| Andorra \| \| --- \| \| Aruba \| \| Australia \| \| Austria \| \| Bahamas, The \| \| Bahrain \| \| Belgium \| \| Bermuda \| \| Brunei \| \| Canada \| \| Cayman Islands \| \| Channel Islands \| \| Cyprus \| \| Denmark \| \| Faeroe Islands \| \| Finland \| \| France \| \| French Polynesia \| \| Germany \| \| Greece \| \| Greenland \| \| Guam \| \| Hong Kong, China \| \| Iceland \| \| Ireland \| \| Israel \| \| Italy \| \| Japan \| \| Korea, Rep. \| \| Kuwait \| \| Liechtenstein \| \| Luxembourg \| \| Macao, China \| \| Monaco \| \| Netherlands \| \| Netherlands Antilles \| \| New Caledonia \| \| New Zealand \| \| Northern Mariana Islands \| \| Norway \| \| Portugal \| \| Qatar \| \| San Marino \| \| Singapore \| \| Slovenia \| \| Spain \| \| Sweden \| \| Switzerland \| \| Taiwan, China \| \| United Arab Emirates \| \| United Kingdom \| \| United States \| \| Virgin Islands (U.S.) \| | \| Australia \| \| --- \| \| Austria \| \| Belgium \| \| Canada \| \| Denmark \| \| Finland \| \| France \| \| Germany \| \| Greece \| \| Hong Kong, China \| \| Iceland \| \| Israel \| \| Italy \| \| Japan \| \| Korea, Rep. \| \| Kuwait \| \| Netherlands \| \| New Zealand \| \| Norway \| \| Portugal \| \| Singapore \| \| Slovenia \| \| Spain \| \| Sweden \| \| Taiwan, China \| \| United Arab Emirates \| \| United Kingdom \| \| United States \| | 98% |

*Example calculation for regional RRT prevalence*

Regional RRT prevalence (eq1)

Population of Country 1 as proportion of total region * Prevalent RRT (HD+PD+Tx [pmp]) in Country 1 + Population of Country 2 as proportion of total region * Prevalent RRT (pmp) in Country 2 … = Total regional prevalent RRT

- Example Region: East Asia Pacific
- Prevalent RRT in China (33 pmp) * Population of China as proportion of total region with available data (0.71) + Prevalent RRT in Indonesia (21.5 prevalent) * Population of Indonesia as proportion of total region with available data (0.12) … = 54 pmp
