# Supplementary material for: The Gap between Estimated Incidence of End-Stage Renal Disease and Use of Therapy
Source: PLoS One. 2013 Aug 30;8(8):e72860. doi: 10.1371/journal.pone.0072860 (PMC3758352; doi:10.1371/journal.pone.0072860)
Supplement: Appendix S3 — Individual country data for incident ESRD in patients with diabetes and hypertension. (DOCX) [file pone.0072860.s003.docx]

**Appendix S3. Individual country data for incident ESRD in patients with diabetes and hypertension**

| **Country** | **Estimated incident ESRD in patients with diabetes (pmp)**  **[range of estimate]** | **Estimated incident ESRD in patients with hypertension (pmp)**  **[range of estimate]** |
| --- | --- | --- |
| Afghanistan | 160 [132, 194] | 78 [74, 80] |
| Albania | 89 [74, 108] | 110 [98, 116] |
| Algeria | 79 [66, 96] | 114 [101, 120] |
| American Samoa | - | - |
| Andorra | - | - |
| Angola | 97 [80, 118] | 102 [92, 106] |
| Antigua and Barbuda | - | - |
| Argentina | 97 [80, 118] | 78 [73, 80] |
| Armenia | 74 [61, 90] | 159 [135, 172] |
| Australia | 61 [51, 74] | 86 [80, 89] |
| Austria | 102 [85, 124] | 96 [88, 101] |
| Azerbaijan | 107 [88, 130] | 140 [120, 150] |
| Bahamas, The | 114 [94, 138] | 93 [85, 97] |
| Bahrain | 105 [87, 127] | 109 [97, 114] |
| Bangladesh | 114[94, 138] | 79 [74, 81] |
| Barbados | 89 [74, 108] | 101 [92, 106] |
| Belarus | 74 [61, 89] | 114 [101, 121] |
| Belgium | 74 [62, 90] | 92 [84, 96] |
| Belize | 59 [49, 71] | 87 [83, 93] |
| Benin | 120 [99, 145] | 176 [146, 191] |
| Bermuda | - | - |
| Bhutan | 85 [71, 104] | 92 [85, 96] |
| Bolivia | 98 [81, 119] | 78 [74, 80] |
| Bosnia and Herzegovina | 67 [55, 81] | 133 [116, 142] |
| Botswana | 92[76, 111] | 182 [151, 199] |
| Brazil | 108 [89, 131] | 165 [138, 179] |
| British Virgin Islands | - | - |
| Brunei Darussalam | 91 [75, 110] | 79 [74, 81] |
| Bulgaria | 75 [62, 91] | 114 [101, 120] |
| Burkina Faso | 52 [43, 63] | 101 [91, 105] |
| Burundi | 40 [33, 48] | 109 [98, 115] |
| Cambodia | 79 [65, 96] | 80 [75, 82] |
| Cameroon | 95 [78, 115] | 121 [107, 129] |
| Canada | 134 [111, 163] | 77 [72, 79] |
| Cape Verde | 62 [52, 76] | 215 [173, 237] |
| Central African Republic | 75 [62, 91] | 100 [90, 104] |
| Chad | 97 [80, 117] | 111 [99, 117] |
| Chile | 90 [74, 109] | 113 [101, 120] |
| China | 182 [151, 221] | 192 [157, 211] |
| Hong Kong SAR, China | 79 [65, 95] | 78 [74, 81] |
| Macao SAR | 56 [46, 68] | 76 [72, 78] |
| Colombia | 67 [55, 81] | 125 [110, 133] |
| Comoros | 68 [57, 83] | 109 [98, 115] |
| Congo DR | 177 [146, 215] | 110 [98, 116] |
| Congo, Rep. | 88 [73, 107] | 119 [105, 126] |
| Cook Islands | - | - |
| Costa Rica | 90 [75, 110] | 87 [81, 91] |
| Cote d'Ivoire | 103 [85, 125] | 134 [116, 143] |
| Croatia | 85 [70, 103] | 126 [110, 134] |
| Cuba | 101 [83, 122] | 87 [81, 90] |
| Cyprus | 68 [57, 83] | 94[86, 98] |
| Czech Republic | 56 [47, 68] | 165 [138, 179] |
| Denmark | 76 [62, 92] | 96 [87, 100] |
| Djibouti | 85 [70, 103] | 99 [90, 103] |
| Dominican Republic | 70 [58, 85] | 98 [89, 102] |
| Ecuador | 80 [66, 97] | 82 [76, 84] |
| Egypt, Arab Rep. | 61 [51, 74] | 107 [96, 112] |
| El Salvador | 89 [74, 108] | 78 [73, 80] |
| Equatorial Guinea | 80 [66, 97] | 106 [95, 111] |
| Eritrea | 66 [54, 80] | 102 [92, 107] |
| Estonia | 71 [58, 86] | 128 [112, 137] |
| Ethiopia | 63 [52, 77] | 117 [103, 123] |
| Fiji | 118 [97, 143] | 103 [93, 108] |
| Finland | 87 [72, 106] | 162 [136, 176] |
| France | 60 [50, 73] | 126 [109, 135] |
| French Polynesia | 162 [134, 196] | 87 [80, 90] |
| Gabon | 80 [66, 97] | 141 [121, 151] |
| Gambia, The | 84 [70, 102] | 122 [108, 130] |
| Georgia | 95 [79, 116] | 114 [102, 121] |
| Germany | 83 [69, 101] | 145 [124, 157] |
| Ghana | 88 [73, 107] | 166.48 [140, 181] |
| Greece [Hellenic Republic] | 82 [68, 99] | 95 [87, 99] |
| Greenland | - | - |
| Grenada | 96 [80, 117] | 93 [85, 97] |
| Guatemala | 95 [78, 115] | 85 [79, 88] |
| Guinea | 77 [64, 94] | 111 [99, 117] |
| Guinea-Bissau | 74 [61, 90] | 106 [95, 111] |
| Guyana | 93 [77, 113] | 85 [79, 88] |
| Haiti | 82 [68, 100] | 86 [80, 89] |
| Honduras | 74 [61, 90] | 88 [81, 91] |
| Hungary | 87 [72, 105] | 134 [116, 143] |
| Iceland | 96 [79, 116] | 94 [86, 98] |
| India | 100 [83, 121] | 111[100, 117] |
| Indonesia | 57 [47, 69] | 114 [102, 120] |
| Iran, Islamic Rep. | 83 [69, 101] | 149 [127, 160] |
| Iraq | 109 [90, 132] | 85 [79, 88] |
| Ireland | 73 [60, 88] | 168 [140, 183] |
| Israel | 87 [72, 105] | 94 [86, 98] |
| Italy | 74 [61, 90] | 158 [133, 171] |
| Jamaica | 87 [72, 105] | 90 [83, 93] |
| Japan | 61 [50, 74] | 176 [145, 192] |
| Jordan | 156 [129, 190] | 97 [88, 101] |
| Kazakhstan | 102 [84, 123] | 102 [92, 107] |
| Kenya | 66 [55, 80.28] | 115 [102, 121] |
| Kiribati | - | - |
| Korea, Dem. Rep. | 143 [119, 174] | 73 [69, 74] |
| Korea, Rep. | 96 [79, 116] | 78 [73, 80] |
| Kuwait | 79 [66, 96] | 83 [78, 86] |
| Kyrgyz Republic | 88 [73, 107] | 123 [108, 130] |
| Lao PDR | 111 [92, 135] | 78 [73, 80] |
| Latvia | 71 [59, 86] | 112 [100, 118] |
| Lebanon | 71 [58, 86] | 89 [82, 92] |
| Lesotho | 121 [100, 147] | 104 [94, 109] |
| Liberia | 97 [81, 118] | 182 [151, 199] |
| Libya | 88 [73, 107] | 138 [119, 147] |
| Lithuania | 83 [68, 100] | 117 [104, 124] |
| Luxembourg | 70 [58, 85] | 87 [80, 90] |
| Macedonia, FYR | 56 [46, 68] | 131 [114, 140] |
| Madagascar | 101 [83, 122] | 204 [166, 224] |
| Malawi | 68 [56, 82] | 172 [144, 187] |
| Malaysia | 80 [66, 97] | 78 [74, 80] |
| Maldives | 103 [85, 125] | 90 [83, 93] |
| Mali | 217 [180, 264] | 96 [88, 100] |
| Malta | 65 [54, 79] | 100 [90, 104] |
| Marshall Islands | - | - |
| Mauritania | 115 [95, 138] | 98 [89, 102] |
| Mauritius | 121 [100, 146] | 175 [146, 191] |
| Mexico | 81 [67, 98] | 84 [78, 87] |
| Micronesia, Fed. Sts. | 96 [79, 116] | 92 [85, 96] |
| Moldova | 80 [66, 97] | 227 [181, 251] |
| Mongolia | 91 [75, 110] | 111 [99, 117] |
| Montenegro | 68 [56, 82] | 111 [99, 117] |
| Morocco | 52 [43, 63] | 103 [93, 108] |
| Mozambique | 71 [58, 86] | 125 [109, 132] |
| Myanmar | 110 [91, 133] | 90 [83, 94] |
| Namibia | 84 [79, 102] | 147 [126, 158] |
| Nauru | - | - |
| Nepal | 117 [97, 142] | 100 [91, 104] |
| Netherlands | 96 [79, 116] | 84 [78, 87] |
| Netherlands Antilles | - | - |
| New Zealand | 66 [55, 80] | 78 [73, 80] |
| Nicaragua | 68 [56, 82] | 108 [96, 113] |
| Niger | 91 [76, 111] | 189 [155, 207] |
| Nigeria | 123 [102, 149] | 156 [132, 168] |
| Norway | 104 [86, 126] | 114 [101, 121] |
| Occupied Palestinian Territory | - | - |
| Oman | 188 [156, 228] | 103 [93, 108] |
| Pakistan | 97 [80, 118] | 83 [78, 86] |
| Palau | - | - |
| Panama | 93 [77, 113] | 110 [98, 116] |
| Papua New Guinea | 51 [42, 62] | 68 [65, 69] |
| Paraguay | 57 [47, 70] | 111 [99, 117] |
| Peru | 71 [59, 87] | 84 [78, 86] |
| Philippines | 65 [54, 79] | 101 [91, 106] |
| Poland | 139 [115, 168] | 151 [128, 162] |
| Portugal | 104 [86, 126] | 114 [101, 121] |
| Puerto Rico | 56 [46, 68] | 112 [99, 118] |
| Qatar | 138 [114, 167] | 146 [125, 157] |
| Romania | 88 [73, 107] | 105 [95, 111] |
| Russian Federation | 53 [44, 64] | 151 [129, 163] |
| Rwanda | 115 [95, 140] | 110 [98, 115] |
| Sao Tome and Principe | 88 [73, 107] | 219 [176, 242] |
| Saudi Arabia | 116 [96, 140] | 125 [110, 133] |
| Senegal | 181 [150, 219] | 105 [95, 110] |
| Serbia | 80 [66, 97] | 133 [115, 142] |
| Seychelles | - | - |
| Sierra Leone | 79 [65, 96] | 248 [196, 276] |
| Singapore | 90 [75, 110] | 111 [99, 117] |
| Slovak Republic | 115 [95, 140] | 106 [96, 112] |
| Slovenia | 78 [65, 95] | 109 [97, 114] |
| Solomon Islands | 67 [56, 82] | 88 [82, 91] |
| Somalia | 91 [75, 110] | 108 [97, 113] |
| South Africa | 92 [76, 111] | 244 [193, 272] |
| Spain | 147 [121, 178] | 130 [112, 139] |
| Sri Lanka | 68 [56, 82] | 106 [95, 111] |
| St. Kitts and Nevis | - | - |
| St. Lucia | 92 [76, 112] | 92 [84, 96] |
| St. Vincent and the Grenadines | 83 [68, 100] | 92 [84, 96] |
| Sudan | 75 [62, 91] | 104 [94, 109] |
| Suriname | 10 [84, 123] | 88 [82, 92] |
| Swaziland | 76 [63, 92] | 106 [96, 112] |
| Sweden | 70 [58, 85] | 127 [110, 135] |
| Switzerland | 79 [65, 96] | 100 [90, 104] |
| Syrian Arab Republic | 113 [94, 138] | 90 [83, 93] |
| Taiwan | - | - |
| Tajikistan | 90 [74, 109] | 100 [90, 104] |
| Tanzania | 63 [52, 77] | 120 [106, 127] |
| Thailand | 54 [45, 66] | 84 [78, 86] |
| Timor-Leste | 78 [64, 94] | 77 [72, 79] |
| Togo | 147 [121, 178] | 108 [97, 114] |
| Tonga | 103 [85, 125] | 108 [97, 114] |
| Trinidad and Tobago | 101 [83, 122] | 94 [86, 98] |
| Tunisia | 88 [73, 107] | 106.87 [96.06, 112.28] |
| Turkey | 104 [86, 126] | 112.84 [100.58, 119.01] |
| Turkmenistan | 58 [48, 70] | 102 [92, 106] |
| Uganda | 89 [74, 108] | 111 [99, 117] |
| Ukraine | 120 [100, 146] | 190 [156, 209] |
| United Arab Emirates | 102 [84, 123] | 95 [87, 100] |
| United Kingdom | 71 [58, 86] | 177 [146, 193] |
| United States | 107 [89, 130] | 98 [89, 102] |
| Uruguay | 89 [73, 107] | 112 [99, 118] |
| Uzbekistan | 108 [89, 131] | 92 [85, 96] |
| Vanuatu | 82 [67, 99] | 138 [119, 148] |
| Venezuela, RB | 97 [80, 118] | 98 [89, 103] |
| Vietnam | 64 [53, 78] | 91 [84, 94] |
| Yemen, Rep. | 96 [80, 117] | 89 [83, 92] |
| Zambia | 63 [52, 77] | 142 [122, 152] |
| Zimbabwe | 74 [62, 90] | 115 [102, 121] |

*Example calculation for incident ESRD due to diabetes*

Incident ESRD (pmp) due to Diabetes in a Single Country (eq1)

DM prevalence* > 20 yr population*Baseline ESRD risk*RR for ESRD in DM = Incident ESRD

Total > 20 yr population

Abbreviations: DM-diabetes, ESRD-end-stage renal disease, RR-relative risk

- Example Country: China
- Prevalence of diabetes in > 20 year old females (0.1) * Total female population > 20 year old (462193882)* Baseline risk for ESRD (13.7 per 100,000 persons-year) * Increase in relative risk of ESRD among patients with diabetes from MrFIT data (12.7) = 45295000
- Total (after adding in males) = 98689560
- Pmp = total (98689560)/ total population for > 20 year old (943225948) = 182 pmp
- Calculation of range: similar procedure, using 10.5 for relative risk for lower limit of range and 15.4 for relative risk for upper limit of range. These relative risks derived from 95% CI for increase in relative risk for ESRD among patients with diabetes in MrFIT.

*Example calculation for incident ESRD due to hypertension*

Incident ESRD (pmp) due to hypertension in a Single Country (eq3)

Baseline ESRD risk*RR for ESRD^[(Mean SBP—Ideal SBP)/Std Dev]*>20 yr population = Incident ESRD

Total > 20 yr population

Abbreviations: ESRD-end-stage renal disease, RR-relative risk, SBP-systolic blood pressure

- Example country: China
- Baseline risk for ESRD (5.3 per 100,000 person-year according to MrFIT data) * Increase in Relative Risk for ESRD (2)^[(Mean SBP in females > 20 years (124.3) – Ideal SBP (115mgHg))/Standard deviation (16)] * Female population > 20 years (462193882) = 70477
- Total (after adding in males > 20 years) = 181,045
- Pmp = total (181,045)/ total population for > 20 year old (943225948) = 192 pmp
- Calculation of range: similar procedure, using 1.8 for relative risk for lower limit of range and 2.1 for relative risk for upper limit of range. 1.8 and 2.1 relative risk derived from 95% CI for increase in relative risk for ESRD among patients with hypertension in MrFIT.

Source of data for prevalence of hypertension:

Danaei, G., et al., National, regional, and global trends in systolic blood pressure since 1980: systematic analysis of health examination surveys and epidemiological studies with 786 country-years and 5·4 million participants. The Lancet, 2011. 377(9765): p. 568-577.

Source of data for prevalence of diabetes:

Danaei, G., et al., National, regional, and global trends in fasting plasma glucose and diabetes prevalence since 1980: systematic analysis of health examination surveys and epidemiological studies with 370 country-years and 2.7 million participants. Lancet, 2011. **378**(9785): p. 31-40.Supplementary Webappendix: pp.1-358.
